# In-Laboratory Polysomnography Worsens Obstructive Sleep Apnea by Changing Body Position Compared to Home Testing

**DOI:** 10.3390/s24092803

**Published:** 2024-04-27

**Authors:** Raquel Chartuni Pereira Teixeira, Michel Burihan Cahali

**Affiliations:** Department of Otolaryngology, Hospital das Clínicas HCFMUSP, Faculdade de Medicina, Universidade de Sao Paulo, Av. Dr. Eneas de Carvalho Aguiar 255, sala 6167, São Paulo 05403-000, SP, Brazil; michel.cahali@hc.fm.usp.br

**Keywords:** home sleep apnea testing, in-laboratory polysomnography, obstructive sleep apnea, apnea–hypopnea index, supine decubitus

## Abstract

(1) Background: Home sleep apnea testing, known as polysomnography type 3 (PSG3), underestimates respiratory events in comparison with in-laboratory polysomnography type 1 (PSG1). Without head electrodes for scoring sleep and arousal, in a home environment, patients feel unfettered and move their bodies more naturally. Adopting a natural position may decrease obstructive sleep apnea (OSA) severity in PSG3, independently of missing hypopneas associated with arousals. (2) Methods: Patients with suspected OSA performed PSG1 and PSG3 in a randomized sequence. We performed an additional analysis, called reduced polysomnography, in which we blindly reassessed all PSG1 tests to remove electroencephalographic electrodes, electrooculogram, and surface electromyography data to estimate the impact of not scoring sleep and arousal-based hypopneas on the test results. A difference of 15 or more in the apnea–hypopnea index (AHI) between tests was deemed clinically relevant. We compared the group of patients with and without clinically relevant differences between lab and home tests (3) Results: As expected, by not scoring sleep, there was a decrease in OSA severity in the lab test, similar to the home test results. The group of patients with clinically relevant differences between lab and home tests presented more severe OSA in the lab compared to the other group (mean AHI, 42.5 vs. 20.2 events/h, *p* = 0.002), and this difference disappeared in the home test. There was no difference between groups in the shift of OSA severity by abolishing sleep scoring in the lab. However, by comparing lab and home tests, there were greater variations in supine AHI and time spent in the supine position in the group with a clinically relevant difference, either with or without scoring sleep, showing an impact of the site of the test on body position during sleep. These variations presented as a marked increase or decrease in supine outcomes according to the site of the test, with no particular trend. (4) Conclusions: In-lab polysomnography may artificially increase OSA severity in a subset of patients by inducing marked changes in body position compared to home tests. The location of the sleep test seems to interfere with the evaluation of patients with more severe OSA.

## 1. Introduction

In-laboratory polysomnography type 1 (PSG1) is the gold standard for the diagnosis of obstructive sleep apnea (OSA). Because PSG1 is assisted by a technician, this test has adequate accuracy and low data loss. However, PSG1 has been considered technically complex and costly [1]. Therefore, given the cost of performing PSG1 in all patients suspected to have OSA and the limited availability of this test in some regions, home sleep apnea testing (HSAT) is a good alternative [2]. HSAT has proved effective in the diagnosis of OSA in areas with a high prevalence of this disease [3,4].

HSAT uses two respiratory variables (effort and flow), in addition to oxygen saturation, and a cardiac variable (heart rate or electrocardiogram), which is commonly known as polysomnography type 3 (PSG3) [2]. PSG3 inevitably underestimates respiratory events in comparison with PSG1 because the denominator of the apnea–hypopnea index (AHI), the main study variable of respiratory events, is the total recording time rather than the total sleep time. Furthermore, PSG3 fails to account for partial respiratory flow reductions (hypopneas) without desaturations, which are scored in PSG1 because they are associated with arousals [5,6]. Approximately 30–50% of patients with OSA awaken very easily with small variations in intrathoracic pressure, destabilizing breathing and preventing the onset of deep sleep. Moreover, without head electrodes for scoring sleep and being in a home environment, which is less “hostile” than the sleep laboratory, patients feel free to move their bodies more naturally to cope with OSA. One study compared different populations matched for age, BMI, AHI, and sex that underwent either PSG1 or PSG3 and concluded that the group in PSG1 stayed in the supine position longer, which could influence the final AHI [7].

In 1984, Cartwright described positional obstructive sleep apnea (POSA) as the presentation of OSA in which supine AHI is at least twice as high as non-supine AHI [8]. Estimates indicate that half of the patients with OSA show worse respiratory parameters in supine decubitus [9,10]. Therefore, changing the supine time between the home and sleep lab could artificially change OSA severity. We aim to verify, in a prospective randomized trial, if PSG1—compared to PSG3—increases AHI due to changes in supine time, regardless of scoring additional hypopneas due to arousals and normalizing AHI by the total sleep time and not total recording time.

## 2. Materials and Methods

This clinical trial was approved by our institution’s Ethics and Research Committee (number 2.954.801/2018) and registered in the Brazilian Research Platform (83077618930010065). STARD reporting guidelines were used [11].

From January 2018 to March 2020, adult (>18 years) patients who visited a private clinic located in the city of Belém, Pará, Brazil, complaining of habitual snoring, daytime sleepiness, and/or sleep apneas reported by the roommate, and with suspected OSA, were prospectively and consecutively included in this study. All patients signed the informed consent form. We excluded those with significant cardiopulmonary disease, neuromuscular disorders, a history of stroke, chronic opioid use, or severe insomnia, those who were unable to understand instructions to perform in-home examinations, and those who had previously performed any sleep tests.

Each patient performed two tests, namely PSG1 and PSG3, on different nights, with a maximum of 7 days from each other. The order of the tests was randomized using the application of the Random Number Generator in GraphPad (San Diego, CA, USA). The following variables were measured: AHI, AHI in the supine position (AHI sup), AHI in the non-supine position (AHI non-sup), minimum oxygen saturation (minimum SpO_2_), time in minutes with SpO_2_ below 90% (T90%), the oxygen desaturation index (ODI, considering a drop of 3% or higher in oximetry), total time in the supine position (TTSP), total sleep time (TST, for PSG1), sleep efficiency (for PSG1), and total recording time (TRT, for PSG3). The denominator for the indexes was TST in PSG1 and TRT in PSG3. We performed an additional analysis, which we termed reduced polysomnography (PSGr), in which we blindly reassessed all PSG1 tests, removing all electroencephalographic (EEG), electrooculogram (EOG), and surface electromyography data to estimate the impact of not scoring sleep and the arousal-based hypopneas on the test results. In PSG1, all indexes (including TTSP) were calculated considering the sleep time, whereas in PSGr and PSG3, all indexes (including TTSP) were calculated considering the total recording time.

PSG1 was performed using the Icelera iBlue 64^®^ system (São Paulo, Brazil), using six EEG channels, EOG, the respiratory effort by thoracic and abdominal plethysmography straps, respiratory flow (nasal cannula and oronasal thermistor), oxygen saturation (pulse oximetry), heart rate, body position (thoracic sensor), leg movement and submental electromyography. Conversely, PSG3 was performed using a Philips Alice PDX^®^ portable sleep diagnostic system (Murrysville, PA, USA), which assesses the respiratory flow (nasal cannula and oral thermistor), respiratory effort using plethysmography straps, body position (thoracic sensor), and oxygen saturation and heart rate (pulse oximetry).

In PSG1, sleep apneas were identified as a drop of at least 90% in the respiratory flow amplitude for 10 s or longer. Hypopneas were defined as a drop between 30 and 90% in respiratory flow amplitude for at least 10 s, associated with a drop of at least 3% in oxygen saturation, or arousal. Sleep apneas were deemed obstructive in the presence of respiratory effort during the event, central in the absence of effort, and mixed in the presence of effort only in part of the event [12].

PSG3 was manually analyzed by the researchers using the same definition for apneas. In PSG3, hypopneas were identified when the respiratory flow amplitude decreased between 30 and 90% for at least 10 s, concurrently with a decrease of at least 3% in oxygen saturation.

According to previous studies, we deemed clinically relevant a difference of at least 15 in the AHI between PSG1 and PSG3 [13,14]. In a post hoc analysis, we compared patients with a clinically relevant difference (Group 1) to those without a clinically relevant difference (Group 2), aiming to identify which factors could explain the difference between these two groups: time in the supine position, AHI supine, arousal-based hypopneas or the normalizing denominator for AHI (TST or TRT).

### Statistical Analysis

We calculated the sample size of 38 patients to achieve a power of 85% for detecting a mean of the differences of 15 in AHI between the tests, assuming the standard deviation of the differences to be 20 [15].

Qualitative variables were analyzed by calculating absolute and relative frequencies. The polysomnographic variables with a non-normal distribution were analyzed by performing non-parametric tests and comparing the results using the Mann–Whitney and Wilcoxon signed-rank tests. Fisher’s exact test was used to compare the prevalence of POSA in Groups 1 and 2. Variables with a normal distribution were compared using Student’s *t*-test.

The agreement between tests was assessed using the intraclass correlation coefficient (ICC) [16]. The magnitude of the agreement estimators (kappa or ICC) was interpreted as follows: 0 (absent), 0–0.19 (poor), 0.20–0.39 (weak), 0.30–0.59 (moderate), 0.60–0.79 (strong), and ≥0.80 (almost complete) [17]. All statistical tests were performed using the software SPSS 17.0 for Windows, setting the significance level at 5%.

## 3. Results

**Figure 1:**
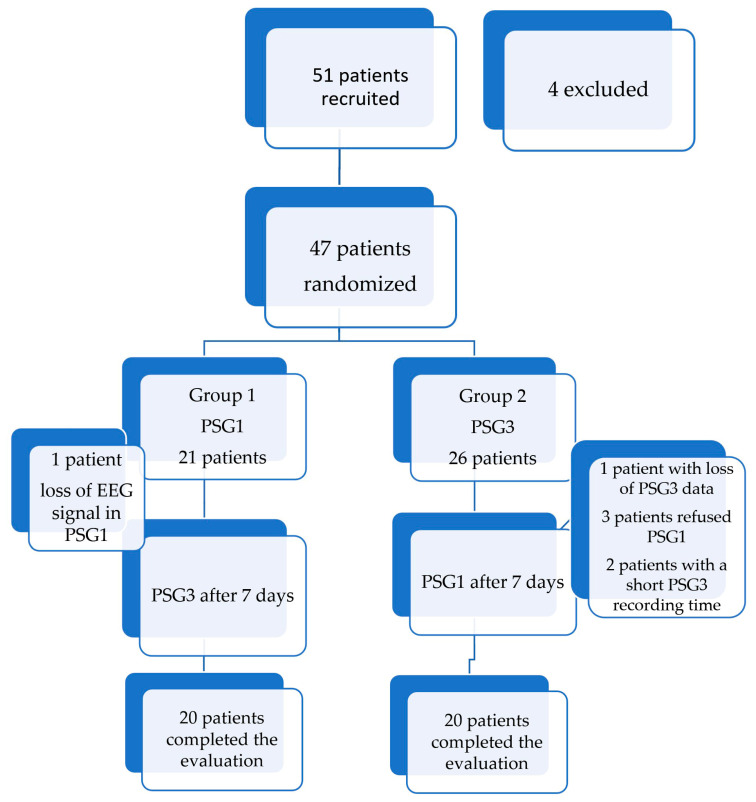
Evaluation protocol flowchart.

In total, 51 patients were recruited for this study, of whom 4 were excluded (one for a history of stroke, two for congestive heart failure, and another for presenting with severe insomnia). A total of 47 patients who met the inclusion criteria agreed to participate in the protocol. Among them, PSG3 data were lost for 1 patient, and another 3 patients declined to undergo PSG1 after the initial PSG3. Two patients were excluded because they used the PSG3 device for less than 2 h, and 1 patient was excluded due to technical problems with the PSG1 electroencephalogram. As a result, 40 patients completed the protocol. In half of the patients, PSG1 was the first test (Figure 1).

Our patients had a mean body mass index (BMI) of 29.1 ± 3.9 kg/m^2^, and seventeen (42.5%) patients were identified as obese. The mean age was 43.7 ± 14.8 years and 30 (75%) were men. Table 1 shows that AHI, AHI sup, and AHI non-sup were significantly higher in PSG1 compared to PSGr. AHI was also higher in PSG1 compared to PSG3. No significant differences were found between the PSGr and PSG3 results for the whole group. Curiously, the mean AHI values were outside the interval between the mean values of AHI sup and AHI non-sup because some individuals predominantly had OSA in the supine position, whereas others predominantly had OSA in the non-supine position (Table 1).

**Figure 2:**
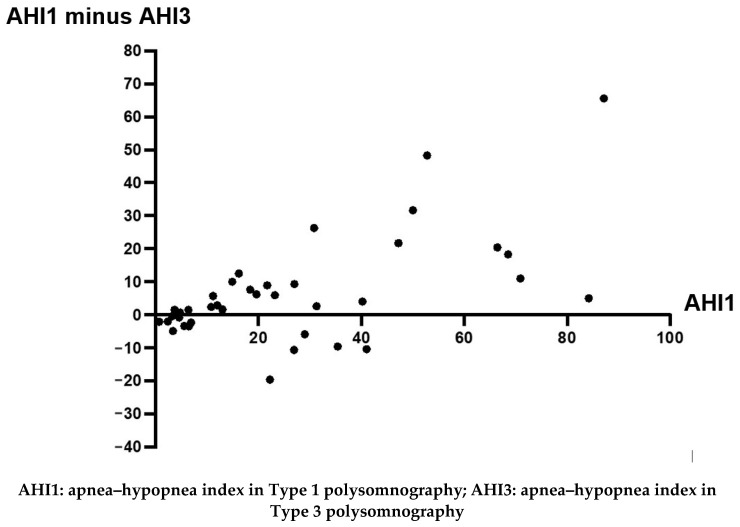
Bland–Altman plot: correlation, in each individual, of the AHI in PSG1 (AHI1) with the difference in AHI values between PSG1 and PSG3 (AHI1 minus AHI3).

In assessing the intraclass correlation coefficient, we found an almost complete agreement of AHI between PSG1 and PSGr (0.80, *p* < 0.0001) and a strong agreement of AHI between PSG1 and PSG3 (0.70, *p* < 0.0001) and between PSGr and PSG3 (0.70, *p* < 0.0001). In terms of severity, in patients with an AHI lower than 30 (mild and moderate OSA), the agreement of AHI between PSG1 and PSG3 was 0.88, and in those with an AHI higher than 30 (severe OSA), this agreement was 0.57 in the intraclass correlation coefficient. In another agreement analysis, the Bland–Altman analysis (Figure 2), we observed that individuals with lower AHI in PSG1 tended to have smaller differences in AHI values between PSG1 and PSG3 (Figure 2).

Table 2 compares patients with (Group 1) and without (Group 2) clinically relevant differences between PSG1 and PSG3. We found no significant differences in age, sex. or body mass index (BMI) between Groups 1 and 2. In PSG1, Group 1 had a significantly more severe OSA and a longer total sleep time than Group 2.

In PSG3, the patients in Group 1 had a higher AHI non-sup than those in Group 2, without significant differences in the other study parameters (Table 3).

The prevalence rates of POSA in Group 1 (66.1%) and Group 2 (37.0%) were similar (*p* = 0.16, Fisher’s exact test). Table 4 outlines the differences between the tests for each group. Because it is important to consider the absolute size of these differences (either an increase or a decrease), we calculated the modulus of the numerical differences for each case in Table 4. By comparing PSG1 and PSG1r, the lack of sleep scoring, particularly arousals, did not account for significant differences between the groups. The differences in AHI sup and in time spent sleeping in the supine position, when comparing in-lab and home sleep tests, were more pronounced in Group 1 than in Group 2, demonstrating the effect of the site of the test on the group that received clinically worst results in the lab test. These variations presented as a marked increase or decrease in supine-related outcomes, with no particular trend.

## 4. Discussion

In our study, in a group of patients with suspected OSA that were prospectively and randomly tested using two sleep tests (in the lab and at home) within a maximum of 7 days apart, AHI was higher in the lab test. As the severity of OSA increased in line with AHI, so did the difference in AHI between the two tests. Patients with a clinically relevant difference between the tests had significantly more severe OSA in the lab but not in the home test. The type (or site) of the sleep test interfered with the evaluation of this group because they presented greater individual variations in total time in the supine position and in AHI in the supine position between the lab and home tests. These variations represented increases or decreases in supine-related outcomes with no particular trend. There was no difference between groups for the shift in OSA severity by abolishing sleep scoring in the lab.

A retrospective study compared a group of patients who experienced PSG1 with other patients who experienced PSG3 and found that the higher the AHI, the more the patient tended to remain in the supine position during PSG1. PSG1 could overestimate AHI due to this greater time in the supine position [7]. Other authors have also suspected that wearing the PSG1 apparatus could increase the time in the supine position in the sleep laboratory, thus overestimating OSA severity [18].

In our study, we did not observe significant differences in the mean time in the supine position between tests, thus corroborating the findings of Kukwa et al., who conducted a retrospective study comparing PSG1 with a home sleep test using WatchPAT™200 (Itamar Medical Ltd., Caesarea, Israel), a portable diagnostic device that monitors peripheral arterial tonometry, oximetry, heart rate, actigraphy, and body position. We need to emphasize that these authors used wireless sensors for electroencephalogram and pulse oximetry monitoring in PSG1, which provides greater freedom of movement for OSA patients [19]. Guerrero et al. found no significant differences in the total time in the supine position between PSG1 and PSG3 for three consecutive nights in a group of subjects with a low pretest probability of OSA [18]. Similarly, Gjevre et al., performing PSG1 and PSG3 with an interval of one week, without randomization, in 47 patients, did not identify a significant difference in total time in the supine position or in AHI sup [20].

We found no difference in AHI supine and in AHI non-supine between PSG1 and PSG3. However, the group that showed OSA to be aggravated in the lab showed greater absolute differences (increase or decrease) in AHI supine between the tests. This unusual finding may be related to the way in which body position is determined in sleep tests. As demonstrated in this study, the thoracic sensor may not be adequate to determine body position. A sensor on the forehead may be more accurate because when the head is turned sideways, even if the thorax is up, the upper airway functions as in the non-supine position [21]. Nevertheless, the other sensors placed on the head in PSG1 could possibly limit the free movement of the head in the lab, whereas, in PSG3, we suppose that the patients could more easily turn their heads into a preferred position that better cope with OSA. Interestingly, after abolishing sleep scoring and arousals, the greater variability in position-related indexes accounted for the worsening of OSA in the group with significant home–lab differences. New wireless technologies that allow comfort and increased mobility to patients during sleep tests may reduce those differences in OSA diagnosis.

The mean AHI was lower in PSG3 than in PSG1. Other authors have already demonstrated that PSG3 tends to present a lower AHI. Berry et al. estimated a mean difference in AHI of 20% between PSG3 and PSG1, with no significant impact on outcomes like adherence to treatment with continuous positive airway pressure (CPAP) and clinical outcomes [5]. The recording time of the entire sample was significantly longer in PSG3 than the sleep time in PSG1, which contributed to a lower AHI because the recording time was the denominator of AHI in PSG3, and the sleep time was the denominator in PSG1. The lack of supervision of a technician did not shorten the recording time in PSG3, as already observed in other studies [22,23].

A finding that caught our attention was that PSG3 showed an overall strong agreement with PSG1 regarding AHI. However, in the Bland–Altman plot analysis, we observed that the difference in AHI between PSG1 and PSG3 tended to increase as AHI increased in PSG1. Currently, the American Academy of Sleep Medicine (AASM) recommends that PSG3 be indicated for patients with a high pre-test probability of moderate-to-severe OSA. In almost all studies used to support this recommendation, the patients had moderate-to-severe OSA, with little data to recommend PSG3 in patients with mild OSA [1]. By contrast, in our study, the outcomes tended to agree more between PSG1 and PSG3 in patients with an AHI lower than 30, thus suggesting that PSG3 is a useful diagnostic tool—particularly for patients with mild and moderate OSA.

A strength of this study is the inclusion of many cases of primary snoring (AHI lower than 5) and mild-to-moderate OSA cases (AHI between 5 and 30). Also, Brazil (including the state of Pará, the site of this study) is a multiracial country with intense miscegenation, which may contribute to the generalization of our outcomes. We believe our study raises confidence at-home sleep tests. Our outcomes should apply to patients with suspected OSA without relevant comorbidities (including insomnia), and that, thus, cannot be generalized.

We need to point out the limitations of home sleep tests. This test seems inadequate for diagnosing OSA in patients with increased sleep fragmentation and without frequent oxyhemoglobin desaturations, such as peri-menopaused women and patients with insomnia. In these patients, the electroencephalogram plays a key role in measuring OSA severity. The effect of frequent arousals cannot be disregarded because they lead to sympathetic hyperactivation, which increases the cardiovascular risk of OSA even without associated desaturations [24].

## 5. Conclusions

In summary, in-lab polysomnography may artificially increase OSA severity in a subset of patients by inducing marked changes in body position compared to home testing.

## Figures and Tables

**Table 1:** Comparison of the data from different polysomnography tests: type 1 (PSG1), reduced type 1 (PSGr, excluding sleep scoring and arousals), and type 3 (PSG3). Mean ± standard deviation.

	PSG1	PSGr	PSG3	*p*-Value PSGr vs. PSG3
AHI (events/h) ^1^	26.9 ± 23.5	23.9 ± 23.7 *	20.9 ±19.1 *	0.92
AHI sup (events/h) ^1^	20.5 ± 22.4	17.8 ± 20.7 *	21.3 ± 22.5	0.28
AHI non-sup (events/h) ^1^	20.6 ± 23.8	19.3 ± 23.8 *	13.8 ± 17.5	0.36
minimum SpO_2_ (%) ^1^	82.6 ± 9.6	82.6 ±9.6	84.0 ± 9.04	0.89
ODI 3% (events/h) ^1^	22.7 ± 30.7	15.3 ± 19.0	17.3 ± 18.5	0.61
T90% (min.) ^1^	12.4 ± 23.1	12.4 ± 23.1	8.11 ± 16.3	0.33
TTSP (min.) ^1^	153.2 ± 112.9	186.6 ± 136.3	186.2 ± 142.9	0.63
TST/TRT (min.) ^2,†^	321.2 ± 63.5	383.6 ± 70.1 *	408.2 ± 56.1 *	0.11

AHI: apnea–hypopnea index; AHI sup: AHI in the supine position; AHI non-sup: AHI in the non-supine position; minimum SpO_2_: minimum oxygen saturation; ODI: oxygen desaturation index; T90%: time under 90% oxygen saturation; TTSP: total time in the supine position; TST: total sleep time; TRT: total recording time; min: minutes. ^1^ Wilcoxon signed-rank tests. ^2^ Student’s *t*-test. ^†^ Total sleep time (PSG1) and total recording time (PSGr and PSG3). * *p* < 0.05 compared to PSG1.

**Table 2:** Type 1 polysomnography (PSG1) data of patients with (Group 1) and without (Group 2) clinically relevant differences between type 1 and type 3 polysomnography tests. Mean ± standard deviation.

Parameter	Group 1 (N = 13)	Group 2 (N = 27)	*p*-Value
Age (years) ^1^	43.4 ± 16.3	43.4 ± 14.9	0.50
BMI (kg/m^2^) ^1^	30.9 ± 5.2	28.6 ± 3.4	0.06
Female (%)	16.7%	29.6%	0.42
AHI (events/h) ^2^	42.5 ± 23.9	20.2 ± 23.2	0.002 *
AHI sup (events/h) ^2^	21.7 ± 39.8	18.2 ± 22.5	0.72
AHI non-sup (events/h) ^2^	27.4 ± 39.8	13.1 ± 20.6	0.46
Minimum SpO_2_ (%) ^2^	74.8 ± 10.8	84.5 ± 8.6	0.02 *
ODI 3% (events/h) ^2^	25.1 ± 23.4	13.1 ± 19.0	0.004 *
T90% (min.) ^2^	15.8 ± 19.0	13.6 ± 18.9	0.04 *
TTSP (min.) ^1^	143.6 ± 126.3	157.5 ± 101.8	0.74
TST (min.) ^1^	352.4 ± 62.0	307.4 ± 60.1	0.04 *
Sleep efficiency (%) ^2^	83.7 ± 11.8	84.5 ± 9.8	0.99

BMI: body mass index; AHI: apnea–hypopnea index; AHI sup: AHI in the supine position; AHI non-sup: AHI in the non-supine position; minimum SpO_2_: minimum oxygen saturation; ODI: oxygen desaturation index; T90%: time under 90% oxygen saturation; TTSP: total time in the supine position; TST: total sleep time; min: minutes. ^1^ Student’s *t*-test for independent samples. ^2^ Mann–Whitney test. * *p* <0.05.

**Table 3:** Type 3 polysomnography (PSG3) data of patients with (Group 1) and without (Group 2) clinically relevant differences between type 1 and type 3 polysomnography tests. Mean ± standard deviation.

Parameter	Group 1 (N = 13)	Group 2 (N = 27)	*p*-Value
AHI (events/h) ^2^	23.6 ± 18.1	18.9 ± 19.5	0.41
AHI sup (events/h) ^2^	28.2 ± 23.2	17.3 ± 22.0	0.10
AHI non-sup (events/h) ^2^	23.8 ± 19.0	12.4 ± 17.8	0.03 *
Minimum SpO_2_ (%) ^2^	77.8 ± 25.5	83.3 ± 9.9	0.84
ODI 3% (events/h) ^2^	14.2 ± 18.8	17.6 ± 18.4	0.40
T90% (min.) ^2^	4.8 ± 9.7	9.8 ± 18.7	0.75
TTSP (min.) ^1^	131.2 ± 92.9	210.6 ± 155.5	0.19
TRT (min.) ^1^	411.7 ± 61.7	406.7 ± 64.6	0.82

AHI: apnea–hypopnea index; AHI sup: AHI in the supine position; AHI non-sup: AHI in the non-supine position; minimum SpO_2_: minimum oxygen saturation; ODI: oxygen desaturation index; T90%: time under 90% oxygen saturation; TTSP: total time in the supine position; TRT: total recording time; min: minutes. ^1^ Student’s *t*-test for independent samples. ^2^ Mann–Whitney test. * *p* < 0.05.

**Table 4:** The absolute size of the differences between the polysomnographic tests: type 1 (PSG1), reduced type 1 (PSGr, excluding sleep scoring), and type 3 (PSG3) in patients with (Group 1), and without (Group 2) clinically relevant differences between in-lab and home tests. Calculated using the modulus of the numerical difference for each case. Mean ± standard deviation ^1^.

Parameter	Difference (between Tests)	Group 1 (N = 13)	Group 2 (N = 27)	*p* Value
AHI (events/h)	PSG1−PSGr	20.0 ± 18.9	2.8 ± 2.9	0.14
	PSGr−PSG3	27.1 ± 21.6	4.2 ± 3.5	0.0003 *
	PSG1−PSG3	23.7 ± 17.8	4.7 ± 3.2	<0.0001 *
AHI sup (events/h)	PSG1−PSGr	6.1 ± 7.2	2.6 ± 3.6	0.28
	PSGr−PSG3	18.4 ± 19.4	8.9 ± 10.2	0.02 *
	PSG1−PSG3	17.9 ± 19.9	9.0 ±10.5	0.01 *
AHI non-sup (events/h)	PSG1−PSGr	4.2 ± 7.9	2.7 ± 7.2	0.31
	PSGr−PSG3	30.9 ± 21.2	6.8 ± 7.7	0.23
	PSG1−PSG3	29.8 ± 20.7	5.4 ± 7.2	0.17
ODI 3% (events/h)	PSG1−PSGr	3.1 (1.4–5.4)	0.7 (0.3–2.6)	0.01 *
	PSGr−PSG3	9.6 (3.6–34.8)	5.0 (2.2–12.3)	0.13
	PSG1−PSG3	11.2 (5.4–20.2)	3.8 (1.2–7.3)	0.007 *
TTSP (min)	PSG1−PSGr	11.8 (5.6–66.9)	22.7 (5.8–50.2)	0.70
	PSGr−PSG3	124.0 (72.5–195.8)	74.0 (31.0–215.0)	0.04 *
	PSG1−PSG3	122.7 (88.7–176.4)	91.5 (45.9–210)	0.04 *
TST/TRT (min)	PSG1−PSGr	62.4 (22.3–104.2)	43.3 (27.6–74.1)	0.44
	PSGr−PSG3	87.0 (45.0–148.8)	76.0 (20.0–112.0)	0.48
	PSG1−PSG3	100.8 (25.4–144.2)	107.1 (58.9–131.7)	0.85

AHI: apnea–hypopnea index; AHI sup: AHI in the supine position; AHI non-sup: AHI in the non-supine position; ODI: oxygen desaturation index; TTSP: total time in the supine position; TST: total sleep time; TRT: total recording time; min: minutes. ^1^ Mann–Whitney test. * *p* < 0.05.

## Data Availability

The raw data supporting the conclusions of this article will be made available by the authors on reasonable request.

## List of Abbreviations

AHI	apnea–hypopnea index
AHI non-sup	AHI in the non-supine position
AHI sup	AHI in the supine position
BMI	body mass index
EEG	electroencephalographic
EOG	electrooculogram
HSAT	home sleep apnea testing
ICC	intraclass correlation coefficient
minimum SpO_2_	minimum oxygen saturation
ODI	oxygen desaturation index
OSA	obstructive sleep apnea
POSA	positional obstructive sleep apnea
PSG1	polysomnography type 1
PSG3	polysomnography type 3
PSGr	reduced polysomnography
T90%	time under 90% oxygen saturation
TRT	total recording time
TST	total sleep time
TTSP	total time in the supine position

## References

[1] Collop N.A. (2007). Clinical Guidelines for the Use of Unattended Portable Monitors in the Diagnosis of Obstructive Sleep Apnea in Adult Patients. J. Clin. Sleep Med..

[2] Kapur V.K., Auckley D.H., Chowdhuri S., Kuhlmann D.C., Mehra R., Ramar K., Harrod C.G. (2017). Clinical Practice Guideline for Diagnostic Testing for Adult Obstructive Sleep Apnea: An American Academy of Sleep Medicine Clinical Practice Guideline. J. Clin. Sleep Med..

[3] Peppard P.E., Young T., Barnet J.H., Palta M., Hagen E.W., Hla K.M. (2013). Increased Prevalence of Sleep-Disordered Breathing in Adults. Am. J. Epidemiol..

[4] Tufik S., Santos-Silva R., Taddei J.A., Bittencourt L.R.A. (2010). Obstructive Sleep Apnea Syndrome in the Sao Paulo Epidemiologic Sleep Study. Sleep Med..

[5] Berry R.B., Hill G., Thompson L., McLaurin V. (2008). Portable monitoring and autotitration versus polysomnography for the diagnosis and treatment of sleep apnea. Sleep.

[6] Kuna S.T., Gurubhagavatula I., Maislin G., Hin S., Hartwig K.C., McCloskey S., Hachadoorian R., Hurley S., Gupta R., Staley B. (2011). Noninferiority of Functional Outcome in Ambulatory Management of Obstructive Sleep Apnea. Am. J. Respir. Crit. Care Med..

[7] Mello A.A.F., D’angelo G., Santos R.B., Bensenor I., Lotufo P.A., Lorenzi-Filho G., Drager L.F., Genta P.R. (2023). Influence of the device used for obstructive sleep apnea diagnosis on body position: A comparison between polysomnography and portable monitor. Sleep Breath..

[8] Cartwright R.D. (1984). Effect of Sleep Position on Sleep Apnea Severity. Sleep.

[9] Heinzer R., Petitpierre N.J., Marti-Soler H., Haba-Rubio J. (2018). Prevalence and characteristics of positional sleep apnea in the HypnoLaus population-based cohort. Sleep Med..

[10] Omobomi O., Quan S.F. (2018). Positional therapy in the management of positional obstructive sleep apnea—A review of the current literature. Sleep Breath..

[11] Bossuyt P.M., Reitsma J.B., Bruns D.E., Gatsonis C.A., Glasziou P.P., Irwig L., Lijmer J.G., Moher D., Rennie D., Kressel H. (2015). STARD 2015: An updated list of essential items for reporting diagnostic accuracy studies. BMJ.

[12] Berry R.B., Budhiraja R., Gottlieb D.J., Gozal D., Iber C., Kapur V.K., Marcus C.L., Mehra R., Parthasarathy S., Quan S.F. (2012). Rules for scoring respiratory events in sleep: Update of the 2007 AASM Manual for the Scoring of Sleep and Associated Events. Deliberations of the Sleep Apnea Definitions Task Force of the American Academy of Sleep Medicine. J. Clin. Sleep Med..

[13] Patil S.P., Ayappa I.A., Caples S.M., Kimoff R.J., Patel S.R., Harrod C.G. (2019). Treatment of adult obstructive apnea with positive airway pressure: An American Academy of Sleep Medicine systematic review, meta- analysis, and grade assessment. J. Clin. Sleep Med..

[14] Roeder M., Bradicich M., Schwarz E.I., Thiel S., Gaisl T., Held U., Malcolm K. (2020). Night-to-night variability of respiratory events in obstructive sleep apnoea: A systematic review and meta- analysis. Thorax.

[15] Dhand N.K., Khatkar M.S. (2014). Statulator: An Online Statistical Calculator. Sample Size Calculator for Comparing Two Paired Means. http://statulator.com/SampleSize/ss2PM.html.

[16] Sim J., Wright C.C. (2005). The Kappa Statistic in Reliability Studies: Use, Interpretation, and Sample Size Requirements. Phys. Ther..

[17] Shrout P.E., Fleiss J.L. (1979). Intraclass correlations: Uses in assessing rater reliability. Psychol. Bull..

[18] Guerrero A., Embid C., Farre R., Duran-Cantolla J., Parra O., Barbé F., Montserrat J.M., Masa J.F. (2014). Management of sleep apnea without high pretest probability or with comorbidities by three nights of portable sleep monitoring. Sleep.

[19] Kukwa W., Migacz E., Lis T., Ishman S.L. (2021). The effect of in-lab polysomnography and home sleep polygraphy on sleep position. Sleep Breath..

[20] Gjevre J.A., Taylor-Gjevre R.M., Skomro R., Frcpc M.D., Reid J., Fenton M., Cotton D. (2011). Comparison of polysomnographic and portable home monitoring assessments of obstructive sleep apnea in Saskatchewan women. Can. Respir. J..

[21] Van Kesteren E.R., Van Maanen J.P., Hilgevoord A.A.J., Laman D.M., de Vries N. (2011). Quantitative Effects of Trunk and Head Position on the Apnea Hypopnea Index in Obstructive Sleep Apnea. Sleep.

[22] Reichert J.A., Bloch D.A., Cundiff E., Votteri B. (2003). Comparison of the NovaSom QCGTM, a new sleep apnea home-diagnostic system, and polysomnography. Sleep Med..

[23] Yin M., Miyazaki S., Ishikawa K. (2006). Evaluation of type 3 portable monitoring in unattended home setting for suspected sleep apnea: Factors that may affect its accuracy. Otolaryngol. Head Neck Surg..

[24] Ucak S., Dissanayake H.U., Sutherland K., Chazal P., Cistulli P.A. (2021). Heart rate variability and obstructive sleep apnea: Current perspectives and novel technologies. J. Sleep Res..

